# Neoadjuvant chemotherapy plus surgery versus concurrent chemoradiotherapy in stage IB2-IIB cervical cancer: A systematic review and meta-analysis

**DOI:** 10.1371/journal.pone.0225264

**Published:** 2019-11-14

**Authors:** Wen Zou, Yiyu Han, Yang Zhang, Chunhong Hu, Yeqian Feng, Haixia Zhang, Jingjing Wang

**Affiliations:** Department of Oncology, The Second Xiangya Hospital of Central South University, Changsha, Hunan, People’s Republic of China; University Hospital Zurich, SWITZERLAND

## Abstract

The optimal treatment strategy for stage IB2-IIB cervical cancer is controversial. This systematic review with meta-analysis evaluated the efficacy of concomitant chemoradiotherapy (CCRT) and neoadjuvant chemotherapy followed by radical surgery (NACT+S). Studies that evaluated NACT+S versus CCRT for patients with Federation of Gynecology and Obstetrics stage IB2-IIB cervical cancer were searched in MEDLINE, EMBASE, and the Cochrane Library database. Hazard ratios (HRs) with their respective 95% confidence intervals (CIs) were calculated using a random-effects model. Toxicity was also evaluated. Six qualified retrospective studies and one randomized controlled trial (2270 patients) were included in this review. The results suggested that compared with CCRT, NACT+S did not improve overall survival in all patients (HR 0.73, 95% CI 0.52–1.02) or stage IIB patients (HR 0.83, 95% CI 0.61–1.15). NACT+S did not improve disease-free survival (DFS) in stage IIB patients (HR 1.10, 95% CI 0.70–1.71). In the analysis of DFS in all patients, a high degree of heterogeneity was detected (I^2^ = 84%). Sensitivity analysis that eliminated these heterogeneous data suggested that CCRT could improve DFS over NACT+S (HR 1.47, 95% CI 1.12–1.93). Diarrhea and rectal and bladder complications occurred at a lower rate in the NACT+S group than in the CCRT group. NACT+S had no survival advantage for patients with stage IB2-IIB cervical cancer compared with CCRT but was associated with fewer side effects. Further prospective studies with a larger sample size of treatment protocols for locally advanced cervical cancer are needed.

## Introduction

Cervical cancer is the third commonest cancer in women worldwide, and, notably, the incidence and mortality rates of cervical cancer are especially high in Asia, Latin America, the Caribbean, and Africa, accounting for approximately 86% of global cervical cancer deaths [1]. It is estimated that over 38% of tumors are diagnosed at International Federation of Gynecology and Obstetrics (FIGO) stage IB2-IIB [2]. However, the treatment strategy for stage IB2-IIB, and especially stage IIB, cervical cancer is controversial. After five large randomized controlled trials in the 1990s [3], cisplatin-based concurrent chemotherapy and external pelvic irradiation followed by brachytherapy (CCRT) has been the preferred treatment option for patients with stage IB2-IIB cervical cancer. Although most patients initially respond to this therapeutic approach, 22%-41% of patients still experience recurrence [4, 5]. Moreover, this treatment is associated with early and long-term toxicities, including radiocystitis, radiation enterocolitis, vaginal stenosis, and pelvic adhesion. Therefore, physicians have been actively exploring more effective treatments. Neoadjuvant chemotherapy (NACT) followed by radical surgery (hysterectomy plus pelvic lymph node dissection) (NACT+S) is the most extensively researched treatment modality and has gained the most attention because it is considered to improve disease control and reduce toxicity.

NACT was first proposed by Feri in 1982 [6], and NACT before surgery and/or radiotherapy for patients with head and neck cancer improves disease-free survival (DFS). After the 1990s, to improve the resection rate of locally advanced cervical cancer, NACT gradually began to be widely administered. Many studies showed that NACT+S could shrink tumors, improve the R0 resection rate, reduce intraoperative spreading risk, reduce the occurrence of postoperative complication, and even improve survival outcomes compared with surgery alone or radiotherapy [7–10]. However, there are disadvantages to this treatment, such as the prolongation of treatment, increased medical expenses, and potential tumor progression caused by insensitivity to chemotherapy [11]. However some studies also indicate that NACT+S has no survival benefit [12]. Now NACT+S remains controversial, especially in the CCRT ear. Further studies have raised the question of which treatment is better for patients with stage IB2-IIB cervical cancer. Therefore, this systematic review with meta-analysis was conducted to compare the clinical outcomes of patients with FIGO stage IB2-IIB cervical cancer receiving NACT+S with those of patients receiving CCRT.

## Methods

### Data sources and search method

Medline, the Cochrane library, Embase were searched for studies published from May 2000 to May 31, 2019. The following MeSH terms and their combinations were searched: “cervical cancer,” “stage IB2-IIB,” “neoadjuvant chemotherapy,” “surgery,” “chemoradiation,” and “radiotherapy.” Reference lists of all recovered trials and relevant reviews were also considered. Some meeting abstracts including the American Society of Clinical Oncology, American Society for Therapeutic Radiology and Oncology, European Society of Gynaecological Oncology, European Conference on Clinical Oncology, and European Society of Medical Oncology were also searched from 2000 to 2019. When some studies have duplicate patient samples, only data from the most recent publication were included in the meta-analysis.

### Study selection

Two researchers (JW and YH) found relevant articles based on the search strategy. The third reviewer (WZ) independently checks the article for possible inclusion and to resolve disagreements.

Studies were included for analysis if they fulfilled the following criteria: (1) cohort or case–control studies that compared NACT+S and CCRT for patients with FIGO stage IB2-IIB cervical cancer; (2) reported the number of patients undergoing both NACT+S and CCRT; and (3) reported overall survival (OS), progression-free survival (PFS), DFS, or adverse reactions in patients undergoing both NACT+S and CCRT. OS was evaluated from the date of randomization to death from any cause or censored at the time of last follow-up. DFS was defined as the time from the date of randomization to the first evidence of clinical recurrence (loco-regional or distant) or death from any cause, or was censored at the date of last follow-up. PFS was defined as the time from the date of randomization until the date of disease progression or death. The definition of PFS was almost identical to that of DFS, with the exception of the survival time of patients with residual tumors. We included articles published in English. We excluded studies with poor literature quality or studies involving patients with stage III-IV. Additional exclusion criteria included lack of original data and incomplete reports. Full-text versions of all eligible studies were obtained for quality assessment and data extraction.

### Data extraction and quality assessment

Data from the included studies were extracted and summarized independently by two investigators (JW and YZ). A third investigator (WZ) was available to resolve discrepancies between the two sets of extracted data. The following data were collected from each study: general identification information (authors, title, journal, date of publication, and duplication of publication), trial, type of patients, intervention characteristics, and reported outcomes. When it was not possible to obtain data from the publication, we tried to contact the authors to provide the information or additional data. Data were directly extracted from the publications or estimated from survival curves using the methods described by Parmar and colleagues [13]. Calculations were carried out using the spreadsheet provided by Tierney and colleagues [14].

The modified checklist based upon the Newcastle-Ottawa scale was used by two investigators (HZ and CH) to assess the quality of the studies. If disagreements were encountered, they were resolved through consultation. This instrument rates observational studies on a nine-point scale based on appropriateness of the study sample, comparability of study groups, and adequacy of assessing exposure and outcomes [15].

### Data synthesis and statistical analysis

Review Manager 5.2 software was used to perform the meta-analysis. For time to event data, the hazard ratio (HR) of the NACT+S arm over the CCRT arm was used as a summary statistic for effect outcomes (OS and PFS), and the 95% CI was calculated for each point estimate. Data were analyzed using the inverse variance method. For dichotomous variables, the effect of treatment was calculated as an odds ratio (OR) and is presented with the corresponding 95% CI. Data were analyzed using the Mantel-Haenszel method. HRs and their respective 95% CIs were calculated using a Der-Simonian and Laird random-effects model [16]. Statistical heterogeneity of the results of the studies was assessed by the chi-square test and expressed with the I^2^ index, as described by Higgins and colleagues [17]. A chi-squared P-value < 0.05 or I^2^ value > 50% were consistent with possible substantial heterogeneity. When heterogeneity was detected, a possible explanation was intensively pursued. If a reasonable cause was found, a separate analysis was then performed. When the cause was not apparent and heterogeneity was caused by divergent data in terms of the direction of the results, we chose not to pool the data. Publication bias was evaluated by Egger’s test [18]. The sensitivity analysis was performed for confirmation of the results when necessary, namely, a single study in the meta-analysis was deleted each time to reflect the influence of the individual data set to the pooled HR.

## Results

### Literature search

The literature search yielded 536 potentially relevant titles. After initial review, 15 titles and abstracts were potentially appropriate. Of these, 7 were excluded for the following reasons: research groups did not match (no comparison between NACT+S and CCRT), the study did not examine the outcome of interest, or the data were insufficient. After reviewing the remaining 8 studies we excluded 1 study that may have included partially overlapping data.

**Fig 1:**
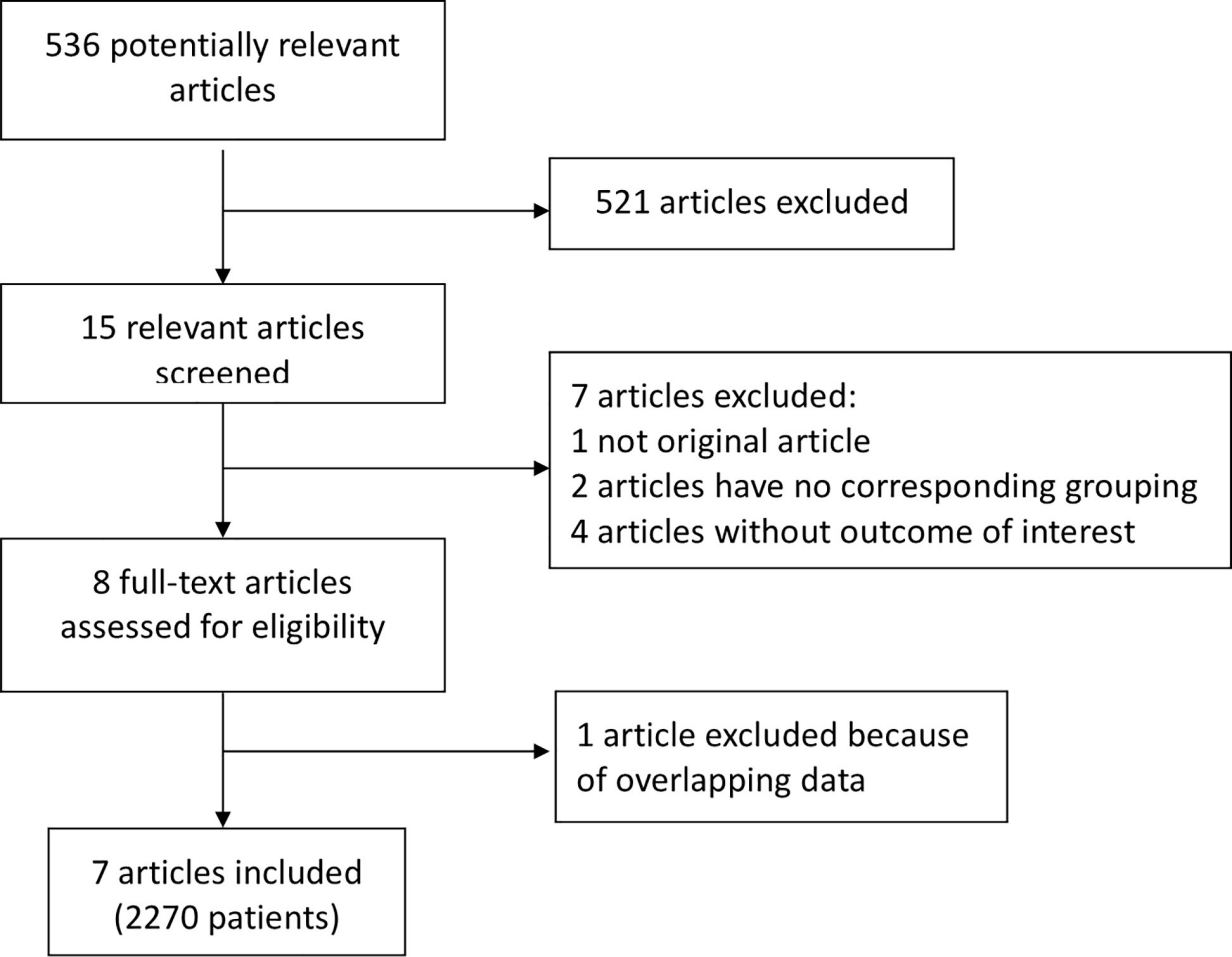
Flowchart showing publication selection.

Finally, 6 qualified retrospective studies and 1 randomized controlled trial, comprising 2270 patients, were included in this review [4, 5, 19–23]. There were 1214 and 1056 patients in the NACT+S and CCRT groups, respectively. Two studies included patients with stage IB2-IIB cervical cancer, 3 included patients with stage IIB cervical cancer, and 2 included patients with stage IB2 cervical cancer. A flowchart shows the detailed process of selection (Fig 1).

### Study characteristics

The main characteristics of the included studies are shown in Table 1. All patients were aged between 20 and 91 years. One of the studies separated people aged <65 and >65 years for prognostic analysis. There were 3 studies that included records of treatment-related toxicity effects, and all studies examined factors influencing OS and DFS. Both NACT and CCRT were platinum-based single-drug or multi-drug combination regimens, in which cisplatin was the main drug. The combination drugs included paclitaxel, VP16, 5-fluorouracil, vincristine, and bleomycin. Patients who were treated with CCRT all received external beam radiotherapy and intracavitary after-loading therapy. Two studies used intensity-modulated radiation therapy for external irradiation; the remaining studies did not describe external irradiation methods in detail. Postoperative supplementary radiotherapy and chemotherapy were administered to the NACT+S group in six studies. In the NACT+S group, 23.1%-96.1% of patients received adjuvant therapy.

**Table 1:** Characteristics of studies identified for the meta-analysis.

References	Journal	Country of origin	Study	Age(median) (years)	Follow-up(median) (months)	Stage	Pathology	Group	Number of Patients (NACT+S/CCRT)	NACT Regimen and Cycle	Patients Received Postoperative Adjuvant Therapy in NACT+S Group	The HR with 95%CI for OS (NACT+S/CCRT)		The HR with 95%CI for DFS (NACT+S/CCRT)		The HR with 95%CI for PFS (NACT+S/CCRT)
Dae Woo Lee et al 2013[13]	International Journal of Gynecological Cancer	korea	retrospective study	25–91	1–139 55	IIB	squamous cell carcinoma	NACT+S group/CCRT group	192 (103/89)	Cisplatin/carboplatin-based chemotherapy 2–3	-	(>60) 24/56 HR:2.22 95%CI : 0.30–16.76	(<60) 79/33 HR:0.63 95%CI : 0.26–1.51	(>60) 24/56 HR:1.65 95%CI : 0.43–6.35	(<60) 79/33 HR:0.94 95%CI : 0.41–2.12	-
Sudeep Gupta et al 2018[5]	Journal of Clinical Oncology	India	Randomized Controlled Trial	26–65	39.3–79.7 (58.5)	IB2-IIB	squamous cell carcinoma	NACT+S group/CCRT group	633 (316/317)	Paclitaxel+ carboplatin 0–3	73 (23.1%)	316/317 HR:1.056 95%CI : 0.773–1.442		316/317 HR:1.46 95%CI : 1.077–1.988	(IIB stage) 179/183 HR:1.9 95%CI : 1.25–2.89	-
ShanShan Yang et al 2015[4]	Tumor Biol	China	retrospective study	25–45 (38)	7–88 (67)	IIB	squamous cell carcinoma	NACT+S group/CCRT group	244 (103/141)	Cisplatin/Nedaplatin/ Carboplatin+Paclitaxel 1–3	65 (63.1%)	103/141 HR:0.85 95%CI : 0.44–1.65				103/141 HR:0.99 95%CI : 0.64–1.54
Lili Guo et al 2015[20]	International Journal of Gynecological Cancer	China	retrospective study	20–65	3.3–130.5	IIB		NACT+S group/CCRT group	621 (285/336)	Cisplatin-based chemotherapy 1–3	274 (96.1%)	283/265 HR:0.85 95%CI : 0.56–1.28		283/265 HR:0.71 95%CI : 0.52–0.97		
He-Yuan Hsieh et al 2018[19]	Journal of the Formosan Medical Association	Taiwan	retrospective study	25–76 (48)	5.6–182.6 (66.2)	IB2		NACT+S group/ S group/CCRT group	66 (39/27)	Cisplatin+vincristine+bleomycin 1–3	16 (41%)	39/27 HR:0.799 95%CI : 0.109–5.837		39/27 HR:2.93 95%CI : 0.831–10.337		
Mingzhu Yin et al 2011[21]	International Journal of Gynecological Cancer	China	retrospective study	23–79	82.8	IB2-IIB		NACT+S group/ S group/CCRT group	281 (187/94)	Cisplatin+ vincristine+bleomycin /cisplatin+paclitaxel 2–3	63 (33.7%)	187/94 HR:0.36 95%CI :0.19–0.68		187/94 HR:0.28 95%CI :0.17–0.46		
H.S. RYU et al 2007[22]	Int J Gynecol Cancer	Korea	retrospective study		0–120	IB2		NACT+S group/CCRT group	(233) 181/52		100(55.25%)	181/52 HR:0.35 95%CI :0.12–1.03				

### Quality assessment

The quality assessment for included studies is described in S1 Table. Of a maximum 9 points, 1 study had a quality score of 5, 1 had a score of 6, 3 had a score of 7, 1 had a score of 8, and 1 had a score of 9. All studies had appropriate cohort selection, including representativeness of the NACT+S cohort and selection of the CCRT cohort. All studies ascertained treatment and stage IB2-IIB cervical cancer patient outcomes through medical records.

### Outcomes: OS, DFS, and toxicity

**Fig 2:**
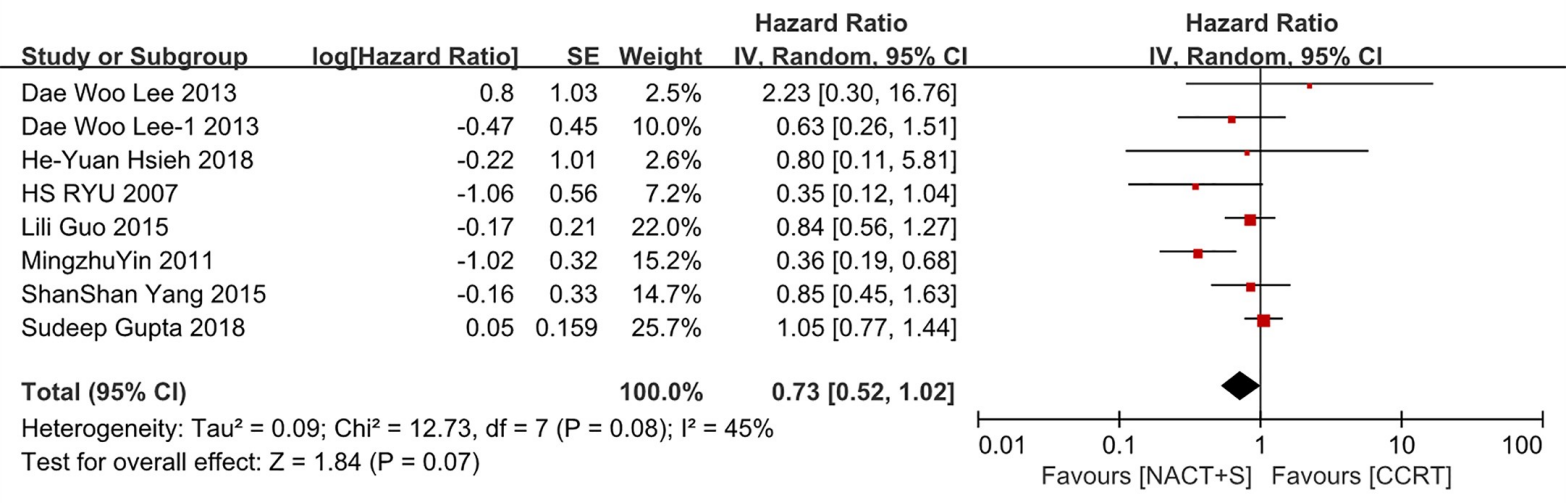
Forest plot of overall survival (OS) for patients with stage IB2-IIB cervical cancer.

OS was analyzed in 7 studies comprising 2270 patients with stage IB2-IIB cervical cancer. The results suggested that NACT+S did not improve OS compared with CCRT in the entire cohort (NACT+S vs. CCRT: HR 0.73, 95% CI 0.52–1.02, P = 0.07), with median heterogeneity among the studies (P = 0.08, I^2^ = 45%; Fig 2). After sensitivity analysis, it was determined that one of the studies was the main cause of the heterogeneity [21], and the heterogeneity was eliminated after its exclusion (P = 0.49, I^2^ = 0%; S1 Fig). However, the results did not change after this exclusion (NACT+S vs. CCRT: HR 0.90, 95% CI 0.72–1.12, P = 0.35). No publication bias was detected using Egger’s test (P = 0.946), and no significant outcome of influence analysis was observed.

**Fig 3:**
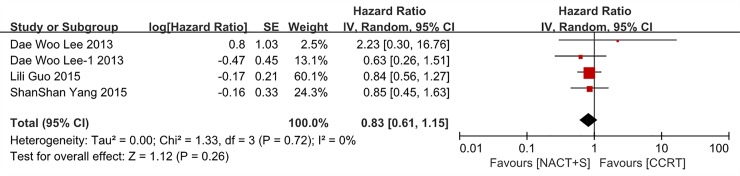
Forest plot of overall survival (OS) for patients with stage IIB cervical cancer.

In a subgroup analysis, OS was analyzed in 3 studies comprising 1085 patients with stage IIB cancer. NACT+S did not improve OS compared with CCRT in this cohort (NACT+S vs. CCRT: HR 0.83, 95% CI 0.61–1.15, P = 0.26), with no heterogeneity among the studies (P = 0.72, I^2^ = 0%; Fig 3).

**Fig 4:**
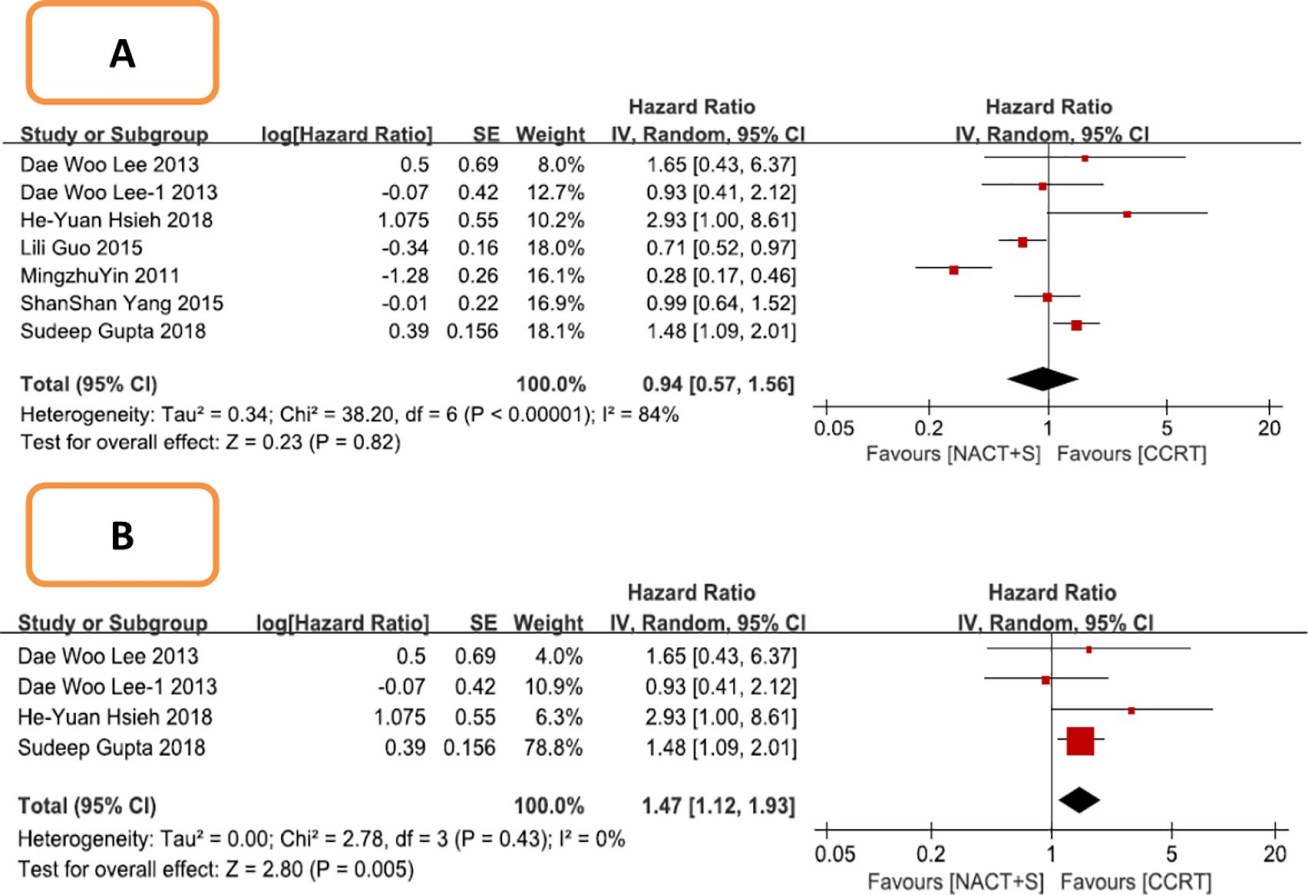
Forest plot of disease free survival (DFS) for patients with stage IB2 IIB cervical cancer. A: Including all studies; B: After excluding three Chinese studies.

DFS was analyzed in 6 studies comprising 2037 patients with stage IB2-IIB cervical cancer. The definition of PFS was almost identical to that of DFS with the exception of the survival time of patients with residual tumors. Therefore, this meta-analysis combined the two results. The results suggested that NACT+S did not improve DFS compared with CCRT among all patients (NACT+S vs. CCRT: HR 0.94, 95% CI 0.57–1.56, P = 0.82); however, high heterogeneity was detected (P < 0.00001, I^2^ = 84%; Fig 4A). Through sensitivity analysis, we found that the heterogeneity was eliminated after excluding all of the Chinese studies (all Chinese studies excluded: P = 0.43, I^2^ = 0%). After this exclusion, we found that CCRT could improve DFS (NACT+S vs. CCRT: HR 1.47, 95% CI 1.12–1.93, P = 0.005; Fig 4B). Moreover, no publication bias was detected with Egger’s test (P = 0.687), and no significant outcome of influence analysis was observed.

**Fig 5:**
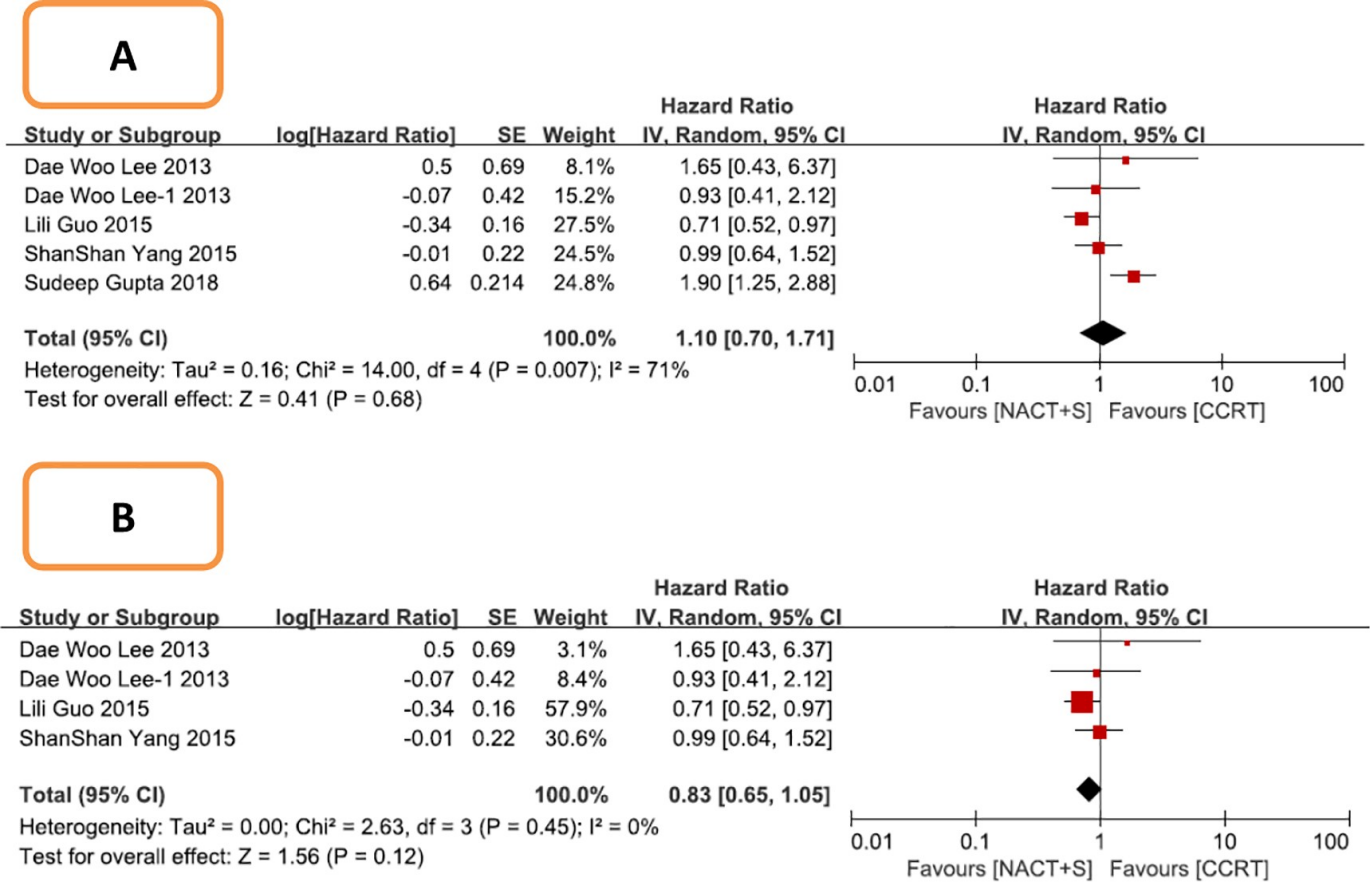
Forest plot of disease free survival (DFS) for patients with stage IIB cervical cancer. A: Including all studies; B: After excluding one Indian study.

In a subgroup analysis, DFS was analyzed in 3 studies comprising 1085 patients with stage IIB cervical cancer. NACT+S did not improve DFS compared with CCRT in this cohort (NACT+S vs. CCRT: HR 1.10, 95% CI 0.70–1.71, P = 0.68), with heterogeneity among the studies (P = 0.007, I^2^ = 71%; Fig 5A). After sensitivity analysis, it was found that the heterogeneity was mainly caused by 1 study, and the heterogeneity was eliminated after its exclusion (P = 0.45, I^2^ = 0%; Fig 5B); this exclusion did not change the result (NACT+S vs. CCRT: HR 0.83, 95% CI 0.65–1.05, P = 0.12).

Toxicity was analyzed in 3 studies comprising 1535 patients with stage IB2-IIB cervical cancer. The early adverse events included hematologic toxicity, nausea, vomiting, diarrhea, and renal failure. The cumulative late adverse events included bladder, bowel, and pelvic and vaginal complications. There was no difference in the incidence of vomiting (OR 1.0, 95% CI 0.74–1.36; S2 Fig) or grade 3 or 4 hematological toxicities (OR 1.69, 95% CI 0.26–11.10, P = 0.58; S3 Fig) between the two groups, whereas diarrhea (OR 0.55, 95% CI 0.31–0.98; S4 Fig), bladder complications (OR 0.27, 95% CI 0.15–0.49; S5 Fig), and rectal complications (OR 0.21, 95% CI 0.13–0.34; S6 Fig) occurred at a lower rate in the NACT+S group than in the CCRT group. All had a high degree of heterogeneity and included fewer studies, and the result may be unreliable.

## Discussion

According to the National Comprehensive Cancer Network guidelines for cervical cancer, CCRT is the most appropriate option for patients with stage IB2-IIB cervical cancer, and other than surgery, it is also the most appropriate option for patients with stage IIA1 disease [24]. However, at present the overall efficacy of CCRT for stage IB2-IIB cervical cancer patients is not ideal [25], and there are some controversies. A meta-analysis in 2002, which used data from 18 trials and 2074 patients, indicated a highly significant reduction in the risk of death with NACT (HR 0.65, 95% CI 0.53–0.80, P = 0.0004) [14]. A multicenter randomized study in Italy also showed a survival benefit of NACT+S compared to conventional radiotherapy (5-year OS, 58.9% vs 44.5%, P = 0.007; 5-year PFS, 55.4% vs 41.3%, P = 0.02) [26]. Although these studies indicated that NACT+S could improve survival, they were all conducted in the era before CCRT treatment.

Compared with CCRT, the efficacy of NACT+S is still controversial and under investigation. The retrospective study results of Lee et al. in 2016 showed that there was no significant difference in survival between stage IB-IIB cervical cancer patients receiving NACT+S and those receiving CCRT [27], and the same results were also suggested in the studies of Singh (stage IB2-IIIA) [28] and Khan (stage IB2 and IIA2) [29]. However, Khan's results indicate that NACT is more advantageous in stage IB2-IIIB patients [30]. Hence, it is urgent to answer this long-standing and important clinical question in the treatment of patients with stage IB2-IIB cervical cancer.

We found only 7 studies that evaluated the efficacy of NACT+S and CCRT in patients with stage IB2-IIB cervical cancer. Some show that NACT+S was more beneficial [20, 21], some found no obvious difference between the two treatment modalities [4, 19, 22], and some indicate that CCRT may have a survival advantage over NACT+S [5, 23]. The results of the meta-analysis showed that NACT+S achieved comparable OS for patients with FIGO stage IB2-IIB cervical cancer compared with CCRT, which was consistent with the results of subgroup analysis in IIB stage.

In terms of OS, the results suggested that NACT+S did not improve OS compared with CCRT in all patients or in stage IIB patients. However, it is important to note that 23.1%-96.1% of patients in the NACT+S group received postoperative adjuvant therapy. Although some studies [31, 32] have shown that adjuvant radiotherapy or chemotherapy is recommended when the patient has certain risk factors after surgery, it can also increase the medical burden and cause other serious complications. In addition, Lee [23] pointed out that some patients who responded well (complete + partial response) to NACT did not have superior OS or DFS than patients who received CCRT. Therefore, CCRT still has advantages over NACT+S.

In the DFS analysis, we found that NACT did not improve DFS in all patients (HR 0.94, 95% CI 0.57–1.56) or in patients with stage IIB disease (HR 1.10, 95% CI 0.70–1.71). However, the results showed obvious heterogeneity, which was still obvious after using the random-effects model. After sensitivity analysis for nationality, adjuvant therapy, synchronous drugs, pathologic type, research method, number of cases, and age, it seemed that the heterogeneity may have been derived from including patients with different nationalities. After the removal of the 3 studies from China, the heterogeneity was eliminated and the results were changed. CCRT improved DFS in all patients (HR 1.47, 95% CI 1.12–1.93), but for stage IIB patients, CCRT did not improve DFS (HR 0.83, 95% CI 0.65–1.05, P = 0.12). Such results may be related to differences in ethnicity, treatment options, and radiotherapy equipment between different countries. Although the initial results suggested that NACT+S is comparable to CCRT in improving DFS, further analysis suggested that CCRT improves DFS over NACT+S.

In terms of toxic effects, 3 studies reported early adverse effects and cumulative late adverse effects of CCRT. Short-term adverse effects were mainly hematologic toxicity and gastrointestinal toxicity caused by chemotherapy or radiotherapy [12, 32]. Long-term adverse effects, such as lymphedema [33] and intestinal and bladder injuries and complications [34, 35], are generally caused by surgery or radiotherapy. After gynecologic oncology treatment (radiotherapy/surgery ± chemotherapy), approximately 40% of patients have gastrointestinal reactions that affect quality of life [36]. Some patients even need surgery to treat urinary tract injury (surgery vs. radiotherapy: 6.3% vs 11.2%) [37]. Our study showed that the NACT+S group appeared to have an advantage in cumulative toxicity compared to the CCRT group. Additionally, radiotherapy can cause ovarian failure in female cancer patients [38, 39]. According to the National Cancer Center, 36.5% of cervical cancer patients are aged <45 years [40]. Therefore, for patients with stage IB2-IIB cervical cancer, NACT+S may be considered as an alternative treatment for young patients who prefer to preserve endocrine function, and this alternative treatment may also be administered when radiotherapy is unavailable.

However, there are still some shortcomings of this study. Six of the included studies were retrospective, and the cycles and drugs of NACT and CCRT were not uniform. We look forward to more large multi-center randomized clinical trials to further confirm the results. Currently, there are ongoing large randomized controlled trials evaluating the efficacy of NACT+S and CCRT. The ongoing European Organization for Research and Treatment of Cancer Trial (EORTC) (ClinicalTrials.gov identifier: NCT00039338) is testing the effect of NACT+S versus that of CCRT in patients with stage IB2-IIB disease using cisplatin-based chemotherapy regimens. Unfortunately, the data are unpublished and therefore could not be included in this meta-analysis. We await additional results from this landmark study to help shed light on the role of NACT+S and CCRT.

## Conclusion

As a systematic review study affected by many factors, our study suggested that NACT+S was not superior to CCRT in terms of survival in stage IB2-IIB cervical cancer, and they can be alternative treatment options. In some respects, CCRT still has advantages. However, patients who are at greater risk of adverse effects or are treated in hospitals without radiological equipment should receive NACT+S. Our conclusions were limited by the retrospective nature of the data. Future prospective studies with a larger sample size are required to confirm our findings.

## Supporting information

S1 Fig Forest plot of OS for patients with stage IB2-IIB cervical cancer after one study eliminated. (PDF)

S2 Fig Forest plot of vomiting. (PDF)

S3 Fig Forest plot of hematotoxicity. (PDF)

S4 Fig Forest plot of diarrhea. (PDF)

S5 Fig Forest plot of bladder complications. (PDF)

S6 Fig Forest plot of rectum complications. (PDF)

S1 Table Quality assessment of studies. (DOCX)

S2 Table PRISMA checklist. (DOC)

## References

[1] Vaccarella S, Laversanne M, Ferlay J, and Bray F. Cervical cancer in Africa, Latin America and the Caribbean and Asia: Regional inequalities and changing trends. INT J CANCER. 2017;141:1997–2001. 10.1002/ijc.30901 28734013

[2] Quinn MA, Benedet JL, Odicino F, Maisonneuve P, Beller U, Creasman WT, et al Carcinoma of the Cervix Uteri. Int J Gynaecol Obstet. 2006;95 Suppl 1:S43–103. 10.1016/S0020-7292(06)60030-1 17161167

[3] Rose PG. Chemoradiotherapy for cervical cancer. EUR J CANCER. 2002;38:270–278. 10.1016/s0959-8049(01)00352-5 11803143

[4] Yang S, Gao Y, and Sun J, Xia B, Liu T, Zhang H, et al Neoadjuvant chemotherapy followed by radical surgery as an alternative treatment to concurrent chemoradiotherapy for young premenopausal patients with FIGO stage IIB squamous cervical carcinoma. Tumour Biol. 2015;36:4349–4356. 10.1007/s13277-015-3074-2 25874487

[5] Gupta S, Maheshwari A, and Parab P, Mahantshetty U, Hawaldar R, Sastri Chopra S, et al Neoadjuvant Chemotherapy Followed by Radical Surgery Versus Concomitant Chemotherapy and Radiotherapy in Patients With Stage IB2, IIA, or IIB Squamous Cervical Cancer: A Randomized Controlled Trial. J CLIN ONCOL. 2018;36:1548–1555. 10.1200/JCO.2017.75.9985 29432076

[6] Frei ER. Clinical cancer research: an embattled species. CANCER-AM CANCER SOC. 1982;50:1979–92. 10.1002/1097-0142(19821115)50:10<1979::aid-cncr2820501002>3.0.co;2-d 7127245

[7] Robova H, Rob L, Halaska MJ, Pluta M, and Skapa P. Review of neoadjuvant chemotherapy and trachelectomy: which cervical cancer patients would be suitable for neoadjuvant chemotherapy followed by fertility-sparing surgery? CURR ONCOL REP. 2015;17:446 10.1007/s11912-015-0446-0 25893880

[8] Angioli R, Plotti F, and Montera R, Aloisi A, Luvero D, Capriglione S, et al Neoadjuvant chemotherapy plus radical surgery followed by chemotherapy in locally advanced cervical cancer. GYNECOL ONCOL. 2012;127:290–6. 10.1016/j.ygyno.2012.07.104 22819938

[9] Peng YH, Wang XX, Zhu JS, and Gao L. Neo-adjuvant chemotherapy plus surgery versus surgery alone for cervical cancer: Meta-analysis of randomized controlled trials. J Obstet Gynaecol Res. 2016;42:128–135. 10.1111/jog.12896 26807961

[10] Kim HS, Sardi JE, and Katsumata N, Ryu HS, Nam JH, Chung HH, et al Efficacy of neoadjuvant chemotherapy in patients with FIGO stage IB1 to IIA cervical cancer: an international collaborative meta-analysis. Eur J Surg Oncol. 2013;39:115–24. 10.1016/j.ejso.2012.09.003 23084091

[11] Iwata T, Miyauchi A, and Suga Y, Nishio H, Nakamura M, Ohno A, et al Neoadjuvant chemotherapy for locally advanced cervical cancer. Chin J Cancer Res. 2016;28:235–240. 10.21147/j.issn.1000-9604.2016.02.13 27199522 PMC4865617

[12] Yang Z, Chen D, and Zhang J, Yao D, Gao K, Wang H, et al The efficacy and safety of neoadjuvant chemotherapy in the treatment of locally advanced cervical cancer: A randomized multicenter study. GYNECOL ONCOL. 2016;141:231–239. 10.1016/j.ygyno.2015.06.027 26115978

[13] Parmar MK, Torri V and Stewart L. Extracting summary statistics to perform meta-analyses of the published literature for survival endpoints. STAT MED. 1998;17:2815–2834. 10.1002/(sici)1097-0258(19981230)17:24<2815::aid-sim110>3.0.co;2-8 9921604

[14] Tierney JF, Stewart LA, Ghersi D, Burdett S, and Sydes MR. Practical methods for incorporating summary time-to-event data into meta-analysis. TRIALS. 2007;8:16 10.1186/1745-6215-8-16 17555582 PMC1920534

[15] Wells G, Shea B OCD, Peterson J, Welch V, and Losos M. The Newcastle–Ottawa Scale (NOS) for assessing the quality if nonrandomized studies in meta-analyses.; 2009, p. Available from: http://www.ohrica/programs/clinical_epidemiology/oxfordasp.

[16] DerSimonian R and Laird N. Meta-analysis in clinical trials. Control Clin Trials. 1986;7:177–188. 10.1016/0197-2456(86)90046-2 3802833

[17] Higgins JP, Thompson SG, Deeks JJ, and Altman DG. Measuring inconsistency in meta-analyses. BMJ. 2003;327:557–560. 10.1136/bmj.327.7414.557 12958120 PMC192859

[18] Egger M, Davey SG, Schneider M, and Minder C. Bias in meta-analysis detected by a simple, graphical test. BMJ. 1997;315:629–634. 10.1136/bmj.315.7109.629 9310563 PMC2127453

[19] Hsieh HY, Huang JW, Lu CH, Lin JC, and Wang L. Definite chemoradiotherapy is a competent treatment option in FIGO stage IB2 cervical cancer compared with radical surgery +/- neoadjuvant chemotherapy. J FORMOS MED ASSOC. 2019;118:99–108. 10.1016/j.jfma.2018.01.015 29429800

[20] Guo L, Liu X, and Wang L, Sun H, Huang K, Li X, et al Outcome of international Federation of gynecology and obstetrics stage IIb cervical cancer from 2003 to 2012: an evaluation of treatments and prognosis: a retrospective study. INT J GYNECOL CANCER. 2015;25:910–918. 10.1097/IGC.0000000000000430 25867278

[21] Yin M, Zhao F, and Lou G, Zhang H, Sun M, Li C, et al The long-term efficacy of neoadjuvant chemotherapy followed by radical hysterectomy compared with radical surgery alone or concurrent chemoradiotherapy on locally advanced-stage cervical cancer. INT J GYNECOL CANCER. 2011;21:92–99. 10.1111/IGC.0b013e3181fe8b6e 21330834

[22] Ryu HS, Kang SB, Kim KT, Chang KH, Kim JW, and Kim JH. Efficacy of different types of treatment in FIGO stage IB2 cervical cancer in Korea: results of a multicenter retrospective Korean study (KGOG-1005). INT J GYNECOL CANCER. 2007;17:132–136. 10.1111/j.1525-1438.2007.00803.x 17291243

[23] Lee DW, Lee KH, Lee JW, Park ST, Park JS, and Lee HN. Is neoadjuvant chemotherapy followed by radical surgery more effective than radiation therapy for stage IIB cervical cancer? INT J GYNECOL CANCER. 2013;23:1303–1310. 10.1097/IGC.0b013e31829da105 23881101

[24] Koh WJ, Abu-Rustum NR, and Bean S, Bradley K, Campos SM, Cho KR, et al Cervical Cancer, Version 3.2019, NCCN Clinical Practice Guidelines in Oncology. J Natl Compr Canc Netw. 2019;17:64–84. 10.6004/jnccn.2019.0001 30659131

[25] Kokka F, Bryant A, Brockbank E, Powell M, and Oram D. Hysterectomy with radiotherapy or chemotherapy or both for women with locally advanced cervical cancer. Cochrane Database Syst Rev. 2015:D10260. 10.1002/14651858.CD010260.pub2 25847525

[26] Benedetti-Panici P, Greggi S, and Colombo A, Amoroso M, Smaniotto D, Giannarelli D, et al Neoadjuvant chemotherapy and radical surgery versus exclusive radiotherapy in locally advanced squamous cell cervical cancer: results from the Italian multicenter randomized study. J CLIN ONCOL. 2002;20:179–188. 10.1200/JCO.2002.20.1.179 11773168

[27] Lee J, Kim TH, Kim GE, Keum KC, and Kim YB. Neoadjuvant chemotherapy followed by surgery has no therapeutic advantages over concurrent chemoradiotherapy in International Federation of Gynecology and Obstetrics stage IB-IIB cervical cancer. J GYNECOL ONCOL. 2016;27:e52 10.3802/jgo.2016.27.e52 27329200 PMC4944019

[28] Singh S, Mishra A, and Goel V, Talwar V., Raina S., Dodagaudar C., et al 2727 Neo-adjuvant chemotherapy followed by surgery vs definitive chemo radiation as treatment for localized carcinoma cervix. EUR J CANCER. 2015;51:S539–540.

[29] İE E, A D, U S, V E, A Ö, and Y Y. The Comparison of Four Different Treatment Modalities in Bulky Stage Ib ve IIa Cervix Cancers. Turk J Obstet Gynecol. 2013;10:42–47.

[30] Khan N. 15th Biennial Meeting of the International Gynecologic Cancer Society. International Journal of Gynecologic Cancer. 2014;24:1. 10.1097/01.IGC.0000457075.08973.89 PMC4230402 25358102

[31] Jung PS, Kim DY, and Lee SW, Park JY, Suh DS, Kim JH, et al Clinical Role of Adjuvant Chemotherapy after Radical Hysterectomy for FIGO Stage IB-IIA Cervical Cancer: Comparison with Adjuvant RT/CCRT Using Inverse-Probability-of-Treatment Weighting. PLOS ONE. 2015;10:e132298. 10.1371/journal.pone.0132298 PMC4503636 26176626

[32] He Y, Zhao Q, and Geng YN, Yang SL, Li XM, Finas D, et al Analysis of short-term efficacy as defined by RECIST and pathological response of neoadjuvant chemotherapy comprised paclitaxel and cisplatin followed by radical surgery in patients with locally advanced cervical cancer: A prospective observational study. Medicine (Baltimore). 2018;97:e10913 10.1097/MD.0000000000010913 29851821 PMC6392635

[33] Kokka F, Bryant A, Brockbank E, Powell M, and Oram D. Hysterectomy with radiotherapy or chemotherapy or both for women with locally advanced cervical cancer. Cochrane Database Syst Rev 2015:D10260. 10.1002/14651858.CD010260.pub2 25847525

[34] Yang Y, Qin T, Zhang W, Wu Q, Yang A, and Xu F. Laparoscopic nerve-sparing radical hysterectomy for bulky cervical cancer (> = 6 cm) after neoadjuvant chemotherapy: A multicenter prospective cohort study. INT J SURG. 2016;34:35–40. 10.1016/j.ijsu.2016.08.001 27519498

[35] Adams E, Boulton MG, and Horne A, Rose PW, Durrant L, Collingwood M, et al The effects of pelvic radiotherapy on cancer survivors: symptom profile, psychological morbidity and quality of life. Clin Oncol (R Coll Radiol). 2014;26:10–7. 10.1016/j.clon.2013.08.003 23992740

[36] Andreyev HJ, Davidson SE, Gillespie C, Allum WH, and Swarbrick E. Practice guidance on the management of acute and chronic gastrointestinal problems arising as a result of treatment for cancer. GUT. 2012;61:179–192. 10.1136/gutjnl-2011-300563 22057051 PMC3245898

[37] Welk B, Wallis C, D'Souza D, McGee J, and Nam RK. A Population-Based Assessment of Urologic Procedures and Operations After Surgery or Pelvic Radiation for Cervical Cancer. INT J GYNECOL CANCER. 2018;28:989–995. 10.1097/IGC.0000000000001266 29664839

[38] Falcone T, Attaran M, Bedaiwy MA, and Goldberg JM. Ovarian function preservation in the cancer patient. FERTIL STERIL. 2004;81:243–257. 10.1016/j.fertnstert.2003.06.031 14967351

[39] Maltaris T, Seufert R, and Fischl F, Schaffrath M, Pollow K, Koelbl H, et al The effect of cancer treatment on female fertility and strategies for preserving fertility. Eur J Obstet Gynecol Reprod Biol. 2007;130:148–155. 10.1016/j.ejogrb.2006.08.006 16979280

[40] National Cancer Institute. Cancer Stat Facts: Cervical Cancer.2016. Available from: https://seer.cancer.gov/statfacts/html/cervix.html.

