# Supplementary figures and images for: Neoadjuvant chemotherapy plus surgery versus concurrent chemoradiotherapy in stage IB2-IIB cervical cancer: A systematic review and meta-analysis
Source: PLoS One. 2019 Nov 14;14(11):e0225264. doi: 10.1371/journal.pone.0225264 (PMC6855659; doi:10.1371/journal.pone.0225264)

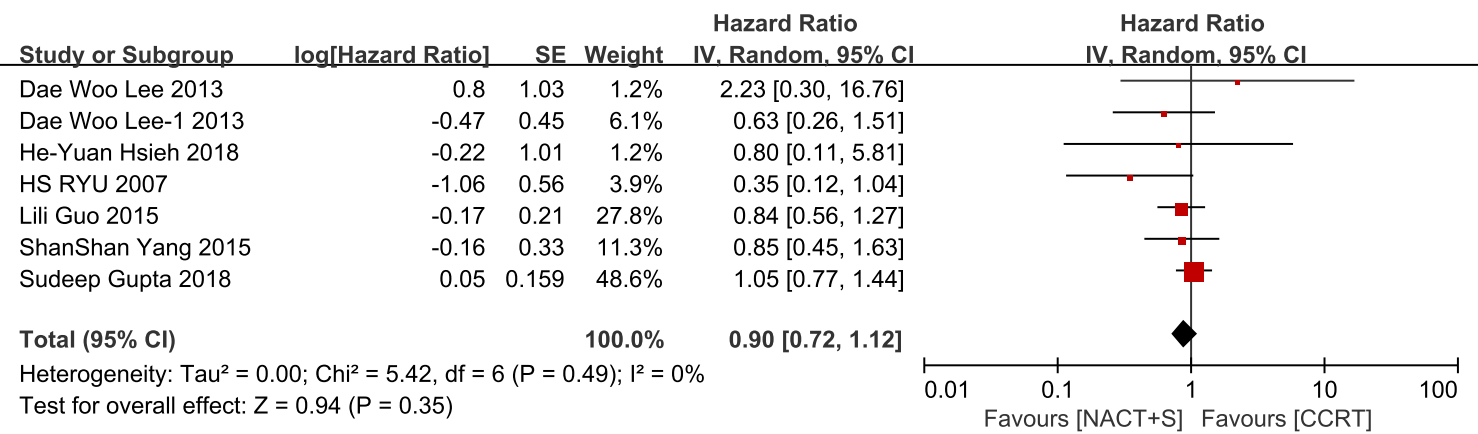

Supplement: S1 Fig — (PDF) [file pone.0225264.s001.pdf]

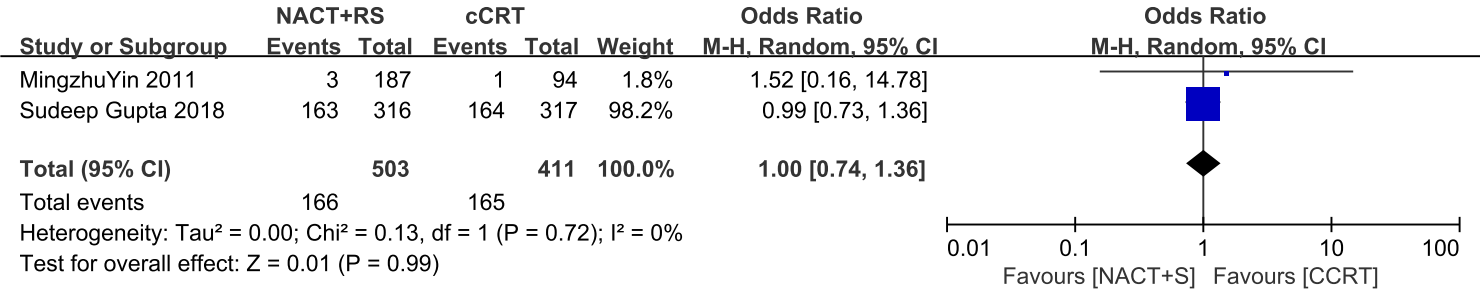

Supplement: S2 Fig — (PDF) [file pone.0225264.s002.pdf]

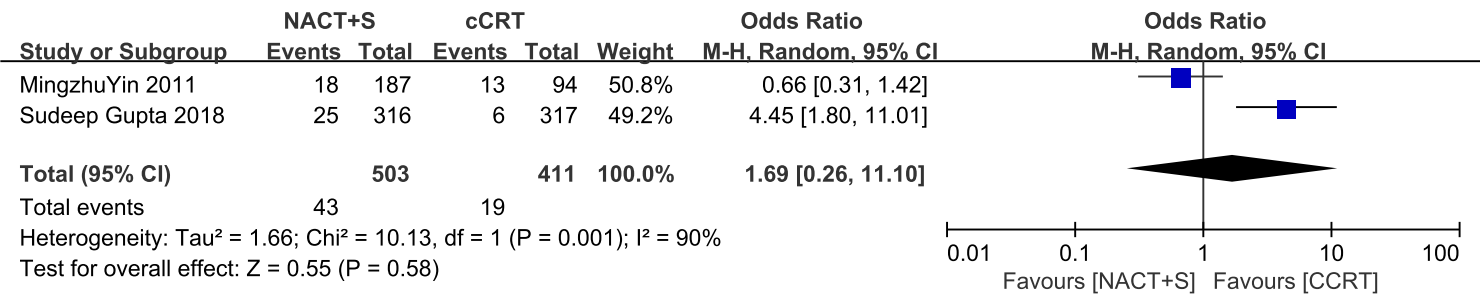

Supplement: S3 Fig — (PDF) [file pone.0225264.s003.pdf]

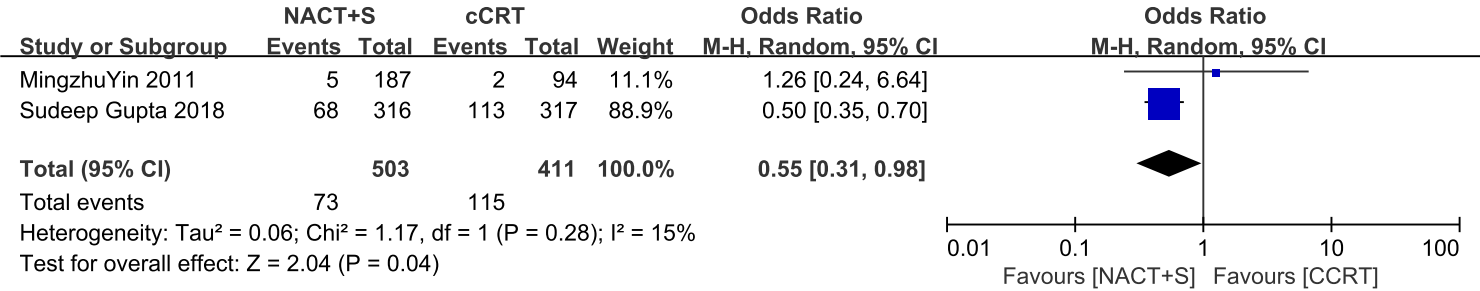

Supplement: S4 Fig — (PDF) [file pone.0225264.s004.pdf]

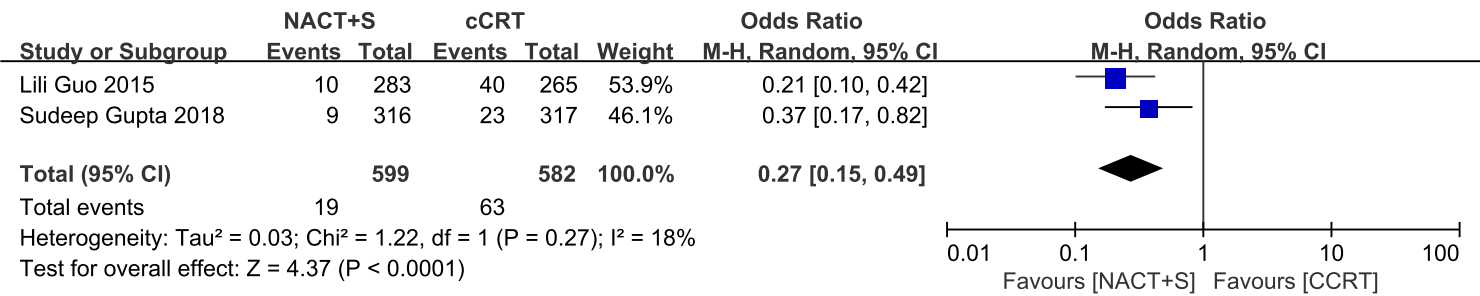

Supplement: S5 Fig — (PDF) [file pone.0225264.s005.pdf]

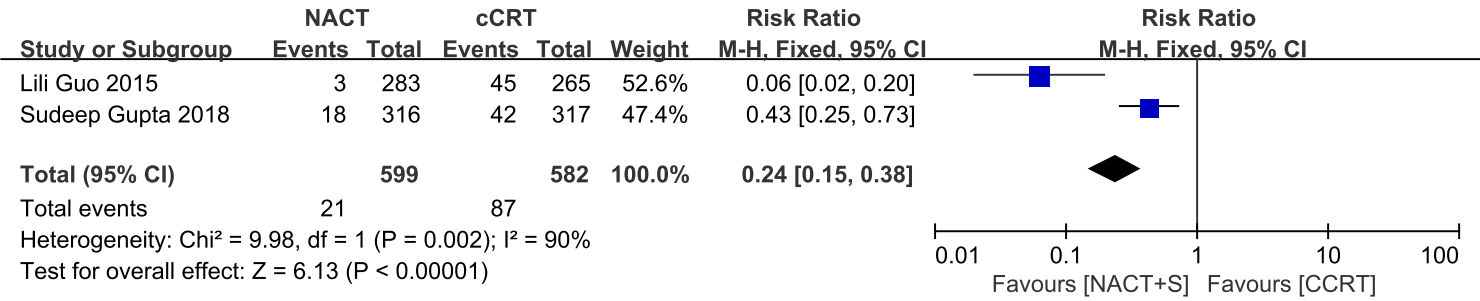

Supplement: S6 Fig — (PDF) [file pone.0225264.s006.pdf]
