# Supplementary material for: Neoadjuvant chemotherapy plus surgery versus concurrent chemoradiotherapy in stage IB2-IIB cervical cancer: A systematic review and meta-analysis
Source: PLoS One. 2019 Nov 14;14(11):e0225264. doi: 10.1371/journal.pone.0225264 (PMC6855659; doi:10.1371/journal.pone.0225264)
Supplement: S1 Table — (DOCX) [file pone.0225264.s007.docx]

S1 Table. Quality assessment of studies

| References | NACT+S Cohort Representative | CCRT Cohort Selection | Ascertainment Of Exposure | Outcome Not Initially Present | Control for Potential Confounders | Assessment of Outcome | Follow-up Period | Follow-up of Cohort | Total score |
| --- | --- | --- | --- | --- | --- | --- | --- | --- | --- |
| Dae Woo Lee et al 2013 | 1 | 1 | 1 | 1 | 1 | 1 | 1 | 1 | 8 |
| Sudeep Gupta et al 2018 | 1 | 1 | 1 | 1 | 2 | 1 | 1 | 1 | 9 |
| ShanShan Yang et al 2015 | 1 | 1 | 1 | 1 | 1 | 1 | 1 | 0 | 7 |
| Lili Guo et al 2015 | 1 | 1 | 1 | 1 | 1 | 1 | 1 | 0 | 7 |
| He-Yuan Hsieh et al 2018 | 1 | 1 | 1 | 1 | 1 | 1 | 1 | 0 | 7 |
| Mingzhu  Yin et al  2011 | 1 | 1 | 1 | 1 | 0 | 1 | 1 | 0 | 6 |
| H.S. RYU et al 2007 | 1 | 1 | 1 | 1 | 0 | 1 | 0 | 0 | 5 |
